# Beyond cultural and geographical proximity: delving into the factors that influence the dynamics of academic relationships between students in higher education

**DOI:** 10.1007/s10734-021-00734-3

**Published:** 2021-07-08

**Authors:** José-Vicente Tomás-Miquel, Jordi Capó-Vicedo

**Affiliations:** 1 Business Administration Department, Universitat Politècnica de València, Plaza Ferrándiz y Carbonell 2, 03801 Alcoy (Alicante), Spain; 2 Economics and Social Sciences Department, Universitat Politècnica de València, Plaza Ferrándiz y Carbonell 2, 03801 Alcoy (Alicante), Spain

**Keywords:** Academic relationships, Proximity effects, University students, Stochastic actor-oriented models (SAOM) of network change, Network dynamics

## Abstract

Scholars have widely recognised the importance of academic relationships between students at the university. While much of the past research has focused on studying their influence on different aspects such as the students’ academic performance or their emotional stability, less is known about their dynamics and the factors that influence the formation and dissolution of linkages between university students in academic networks. In this paper, we try to shed light on this issue by exploring through stochastic actor-oriented models and student-level data the influence that a set of proximity factors may have on formation of these relationships over the entire period in which students are enrolled at the university. Our findings confirm that the establishment of academic relationships is derived, in part, from a wide range of proximity dimensions of a social, personal, geographical, cultural and academic nature. Furthermore, and unlike previous studies, this research also empirically confirms that the specific stage in which the student is at the university determines the influence of these proximity factors on the dynamics of academic relationships. In this regard, beyond cultural and geographic proximities that only influence the first years at the university, students shape their relationships as they progress in their studies from similarities in more strategic aspects such as academic and personal closeness. These results may have significant implications for both academic research and university policies.

## Introduction

Over the last few years, social and academic relationships of university students have received great attention from both literature and practitioners. Universities are crucial contexts for the development of social networks because campus life is for many students the first experience outside the parent-shaped environment (Mayer & Puller, 2008). The resulting social interactions can have far-reaching effects as they allow undergraduates to obtain both personal and academic support inside and outside the classroom, thus helping them cope with the pressures and challenges that often arise in the university context (Gašević et al., 2013; Tomás-Miquel et al., 2016).

The recognition of the importance of these social relations in the educational context together with the development in recent years of analytic techniques in social network analysis (SNA) allows some new and unexplored issues to be addressed, in particular those related to knowledge exchanges, selection and socialisation processes, psychosocial problems and student performance, among others. In this paper, the students’ academic network, as a remarkable factor that influences their performance, has been analysed. To develop our analysis, we have combined proximity factors and a dynamic approach, overcoming past partial or unsatisfactory approaches.

As previously stated, students’ social relationships have been considered for a long time a valuable source of support and assistance. However, as recent literature suggests, not all links between students have the same importance and influence (Chen et al., 2012; Gašević et al., 2013; Tomás-Miquel et al., 2016). Therefore, it is essential to consider not only the structure of the networks that students form but also the content of the links (Smith & Peterson, 2007).

In this regard, the literature identifies two main types of linkages among students (Ibarra, 1993). On the one hand, there are the instrumental or academic ties which are linked to the exchange of knowledge among students. These relationships provide support to the student in the form of sharing notes, solving complex problems and allowing group study (Chen et al., 2012). On the other hand, we can also find expressive or friendship ties which are more linked to affection, emotions and attachment. These cover relationships that involve the exchange of friendship and trust and are not necessarily study-related (Brouwer et al., 2018; Smith & Peterson, 2007).

With regard to academic linkages, despite their relevance and influence on students’ academic performance (Berthelon et al., 2019; Smith & Peterson, 2007), they have not been a major research area in comparison with other types of linkages of a more friendship nature. The reasons for this are not obvious; however, we have found great interest in recent years for the study of the influence of friendly relationships among adolescents on problems such as delinquency, bullying, depression, anxiety, alcohol consumption, smoking or obesity and on socio-cognitive aspects such as personality or morality (Rambaran et al., 2017), which may have ultimately detracted from other research areas such as academic relationships.

In addition to the importance of the typology of links, the mechanisms that influence the dynamics of student relationships have also received significant attention in the literature. Among these mechanisms, past contributions have strongly highlighted homophily, i.e. the tendency of students to affiliate with peers who already have similar behavioural tendencies and attitudes (Kandel, 1978; Lazarsfeld & Merton, 1954; McPherson et al., 2001), as a major factor triggering the formation of relationships among students. Aspects such as similarity in age, gender, race, academic achievement or career aspirations have been shown to be predictive variables in the selection and relationship-building processes between university students (Brouwer et al., 2018; Mayer & Puller, 2008; Thiele, Sauer, Atzmueller, & Kauffeld, 2018a; Ullrich et al., 2010). Additionally, the literature has highlighted geographical proximity as one of the main causes of homophily because spatial closeness increases the likelihood that people that are alike can meet and interact (McPherson et al., 2001; Preciado et al., 2012).

It is also worth noting that according to Rambaran et al. (2017), the study of these aspects has been boosted by the appearance in recent years of analytic advances in SNA such as stochastic actor-oriented model (SAOM), which allow for a more accurate estimation of peer effects (Snijders, 2001, 2005; Snijders et al., 2010).

Although current literature has shed some light on the presence of some similarity factors that favour the formation of links between university students, we consider that there are still some important questions to be addressed in this research area. Some studies have looked at these issues from a longitudinal perspective (Gasevic et al. 2013; Hommes et al., 2014; Rienties & Nolan, 2014). Nonetheless, despite these efforts and the emergence of new analysis techniques, the existing literature has not been able to deepen the understanding of these similarity factors, especially when addressing the whole student’s career at university. From the current body of research, it becomes clear that peers are remarkably similar in a wide range of characteristics. However, in our opinion, there is a problem related to the generalisation of these conclusions. In this vein, the influence of the similarity factors may differ from the early to late years of students at the University. For example, first-year students often do not know their classmates well enough and therefore may be unaware of many of their more personal aspects, such as interests, behaviour, habits and academic performance. On the other hand, students in their final years may have more mature work expectations and objectives as they approach their incorporation into the labour market, as well as greater knowledge and experience with their peers. These facts and changes in behaviour can influence how students establish relationships with their peers, and therefore the similarity factors that prevail among them over time. In fact, recent contributions such as Thiele, Sauer, Atzmueller, and Kauffeld (2018a) encourage to consider in future research the influence of time in the selection processes and formation of links between students.

This work attempts to fill this gap and to contribute to a better understanding of the mechanisms that influence the dynamics of academic relationships between undergraduates by exploring through SAOM and student-level data the influence that a set of social, personal, geographical, cultural and academic proximity factors may have on the formation of these relationships over the entire period in which a student studies at university. In an academic context in higher education that gives increasing importance to collaborative working groups and learning communities (Brouwer et al., 2018; Zhao & Kuh, 2004), this research makes it possible to improve the understanding of how students relate in the classroom and therefore their organisation into efficient and productive working groups. To proceed with the empirical study, we selected students from the bachelor’s degree in Business Administration and Management from the Campus of Alcoy of the Polytechnic University of Valencia in Spain. Specifically, our study focused on the 76 students of the 2016–2020 cohort.

The content of this article has been organised into five sections. Thus, after the introduction, the second section presents the theoretical framework on which the research is grounded, along with the research questions. The next section presents the research setting which encompasses the description of the research context and the sample, the data collection procedure, the analysis techniques and the main variables addressed in the research. The fourth section describes the results of the empirical study. Finally, main conclusions and prescriptions of the research are reported in the last section, jointly with the limitations of the work and future research lines.

## Theoretical background and research questions

A vast body of the literature has confirmed the importance and influence of social relationships at the university on the satisfaction, persistence and especially academic performance of undergraduate students (Berthelon et al., 2019; Gašević et al., 2013; Smith & Peterson, 2007; Tomás-Miquel et al., 2016). Overwhelmed by many fears, insecurities, projects and exams, bonds with other students can serve as a support for coping and overcoming them. On the contrary, social bonds can be also a source of stress, suffering and anger. As a result, the relationships of university students do not remain completely stable during their time at university but tend to evolve over time.

The study of the dynamics of selection among students has received preferential attention from scholars for many decades. In particular, the literature has focused on the mechanisms that regulate the dynamics of friendship relationships, i.e. those factors that make it easier for two students to establish a friendship between them. As previously noted, one of these factors that has attracted the most attention from researchers has been homophily, which claims that contacts between similar people occur more frequently than between dissimilar people (McPherson et al., 2001).

The study of homophily in universities has received increasing attention in recent years thanks to the proliferation and availability of longitudinal network data (Paul & O’Malley, 2013) and the emergence of new analytical techniques within SNA such as SAOM (Snijders, 2005) which have allowed an easier and a more accurate estimation of its influence (Baerveldt et al., 2008).

A review of the literature allows us to find empirical evidence of the presence of diverse homophily factors that influence the formation of academic and social bonds in university environments. In this vein, Mayer and Puller (2008), in a study based on Facebook relationships, found that residential proximity, social proximity (i.e. the existence of common friends between two students), gender, socioeconomic background and race were significant predictors of friendship between university students. In the same way, Marmaros and Sacerdote (2006), from data on email communication between students and recent graduates, also found evidence of this positive influence in establishing friendly relationships for the variables race, geographical closeness, family background and common interests. In a context of friendship relationships and Twitter interactions, Ullrich et al. (2010) obtained empirical evidence of the influence of gender in the formation of relationships among university students. On the other hand, in a mixed context of friendship and academic relationships, Thiele, Sauer, Atzmueller, and Kauffeld (2018a) found evidence of the influence of proximity factors such as age, gender and career aspirations on the formation of bonds among university students. Finally, Brouwer et al. (2018), already in a context of purely academic relationships, also obtained evidence of the influence of gender and academic performance on the formation of bonds among freshman students.

The analysis of this evidence allows us to raise two major points. On the one hand, previous results have found empirical evidence in university contexts of the presence of various proximity factors linked to social, cultural, geographical, academic and personal aspects that favour the formation of links between students. On the other hand, although learning and work relations, as well as inter- and inter-group learning relations in academic relationship-based settings have received notable attention in the literature, for example, in Australia (Neri & Ville, 2008), Spain (Hernandez-Nanclares et al., 2017; Tomás-Miquel et al., 2016), The Netherlands (Hommes et al., 2012; Hommes et al., 2014) and the UK (Héliot et al., 2020; Rienties & Héliot, 2018; Rienties & Tempelaar, 2018), specific studies on homophily or proximity factors in these contexts are still scarce, thus requiring further attention.

On this basis, we focus in this research on the study of the development of academic bonds between university studies. Recent studies such as Chen et al. (2012) with master’s degree students or Brouwer et al. (2018) with freshman students suggest that the dynamics of social and academic relationship formation at university may show similar patterns. However, in our opinion, empirical evidence in this topic is still scarce and needs some degree of extension. Further studies are therefore needed to expand and contrast the influence of the different proximity factors in new contexts, especially, those addressing academic relationships. Hence, with the goal of providing new empirical evidence in this area and, on the basis of the framework of social, personal, geographical, cultural and academic proximity factors previously identified from the literature, we propose our first research question:

Research question 1: *Which proximity factors influence the establishment of academic bonds between university students?*

In parallel, there are other important gaps that in our view still wait to be bridged in this research field. In this regard, it becomes necessary to reflect on the influence that the aforementioned proximity factors can have on the formation of academic links, keeping in mind the whole-time window in which the student is enrolled at university. In this sense, recent studies, aware of this gap, encourage to address its analysis in future contributions (Thiele, Sauer, Atzmueller, & Kauffeld, 2018a). Once students enter university, they undergo a mental evolutionary process that will facilitate their development as people in society. In addition, from that moment onwards, a continuous increase in their relational experience and maturity occurs. The question that most concerns us in this respect is whether this personal evolution will influence the way in which the undergraduate student configures its relationships throughout their time at university. If so, the similarity factors that would influence at the beginning of this stage could differ from those predominant in the final academic years.

Studies among adolescents such as Lubbers et al. (2011) or Preciado et al. (2012) already suggest a divergent influence over time of some proximity variables such as race and gender. However, to our knowledge, we do not find any research in higher education settings that addresses this issue.

Therefore, this paper also aims to fill this gap by contributing to the existing body of knowledge through the study of the influence of the proximity factors on the dynamics of academic links between students throughout their time at university. To this end, two additional research questions are proposed:

Research question 2: *Does the influence of the proximity factors differ throughout the period in which undergraduate students are enrolled at university?*

Research question 3: *If so, is there a pattern that explains this variation?*

## Method

This research is based on the Campus of Alcoy of the Polytechnic University of Valencia in Spain. It offers six bachelor’s degrees, two double degrees and four master’s degrees. As for the university community and according to the 2019/2020 University’s academic annual report, it is composed of 2,326 students. Increasingly, the Campus brings together students from different countries, both European and from other continents, and ethnicities. On the other hand, and concerning the staff, the Campus has 278 employees, 60 of whom work in administration and services and 218 in research and teaching activities.

We are aware that the research context in which the data have been collected may differ from other countries. In this sense, access to university in Spain will be defined by an access mark determined by the average mark of the high school and the qualification of the so-called university access exams in which students are examined on the subjects studied in the last year of the high school. In addition, there is also the possibility of accessing university through other routes, those over 25 years old by means of a specific entrance exam and those over 40 years old by accreditation of work and professional experience. This means that the age distribution of university students can be very diverse. Moreover, admission to university is restricted by *numerus clausus*.

Regarding the performance rate at this specific bachelor’s degree in Business Administration and Management, it is higher than the average for all universities and degrees in Spain (85.95% vs. 77.77%), while the initial drop-out rate (in the first year) is slightly lower than the average (20.97% vs. 21.70%).

This bachelor’s program is characterised by having all compulsory subjects during the first three years, while in the fourth year students must choose between two intensifications with their corresponding optional subjects.

In relation to the learning design and pedagogy employed in the different target modules, they are mostly based on the use of active learning methodologies in a collaborative context of teamwork among students. This scenario allows students to find multiple opportunities to interact both in the classroom context by collaborating in activities to jointly solve problems or doubts, develop tasks or undertake practical experiments, and outside the classroom by collaborating in the development of joint projects and activities.

### Participants and data collection

As we have mentioned above, our sample is composed by students of the bachelor’s degree in Business Administration and Management. This bachelor’s degree is a four-year full-time program requiring the completion of 240 ECTS (European Credit Transfer System) credits which aims to train future professionals able to manage, run, advise and assess business organisations.

In this research, we have focused on students who have completed their first three years at university (2016–2020 cohort) because of three main reasons. Firstly, these students have been able to extensively develop their relationships in this context after three academic years. On the other hand, in the fourth year, a large part of the students decides to carry out part of their studies in foreign universities mainly thanks to the scholarships of the European Erasmus+ Programme or to carry out internships in companies, which prevents them from adequately developing their relations in the university environment. In addition, we have excluded from the study exchange students coming from other countries to the Campus only for a few months because they are enrolled in subjects from different courses/levels and are not able to extensively develop their relationships in the classroom. Last but not least, the selection of these students was also determined by the fact that during the first three years in the selected bachelor’s degree all courses are mandatory. This prevented the possibility of course selection available to students in the fourth year from having an influence on the formation of academic links.

The total number of students considered in this bachelor’s degree was 76. However, this number was smaller in the first year, since five full-time new Spanish students enrolled in the bachelor’s degree in the second year. Apart from this change, the composition of the students’ cohort has been the same for the three years analysed, which makes the sample extremely appropriate for studying the research questions raised.

Concerning data collection, the information provided by the students of the 2016–2020 cohort during their first three years at the University was the main data source of this research. Before obtaining information from the students, they were informed about the research objectives, ethical aspects and procedures that we were going to develop during the three years of the study. This was carried out by using a “roster-recall” method (Wasserman & Faust, 1994), which involves presenting to the interviewees a full list of the students in the cohort who were then asked about their academic relationships with each of them. Specifically, each student was asked annually at the end of each academic year (first year in May 2017, second year in May 2018 and third year in May 2019) about the students that he/she had helped that year in the bachelor’s degree to develop projects, exercises and joint classroom activities, as well as to prepare for exams. If student *i* had nominated student *j* in year *k*, we assigned a value of 1 to x_*ijk*_ (i.e. knowledge transfer from student *i* to student *j* in year *k*), zero otherwise. This information allowed us to build three academic relationship networks, one for each academic year. Additionally, students were also asked every year about different personal and academic aspects such as age, residence addresses and academic performance and interests. All this information was complemented with secondary data sources such as projects and joint activities developed in different subjects to increase its validity (Yin, 1989). In particular, it was reviewed by a panel of experts consisting of the academic committee and the lecturers of the bachelor’s degree, who confirmed the veracity of the relationships and the academic data provided by the students. At the end of the data collection procedure, we had 76 valid responses which represents the whole population of the students’ cohort addressed. On this basis, relational data was stored in a set of three 76×76 matrices, one for each academic year. In each matrix, and following the logic presented above, *x*_*ij*_*=*1 indicates a knowledge transfer from student *i* to student *j*, and *x*_*ij*_*=*0 when there is no knowledge transmission from student *i* to student *j*.

### Analytical approach

Individual attributes of the network participants and structural features of the peer networks for each wave were initially described. In addition, with the purpose of analysing the research questions proposed, a SAOM for network dynamics (Snijders, 2001, 2005; Snijders et al., 2010) was applied. In particular, we used Siena (Simulation Investigation for Empirical Network Analysis) (Ripley et al., 2020), a software to statistically estimate models for network evolution. More specifically, we used RSiena, a contributed package to the statistical software environment R. This set of methods has been used extensively in both business and social research contexts. In the educational field, it has already been used successfully in recent research articles such as Dijkstra et al. (2013), Rambaran et al. (2017), Brouwer et al. (2018) or Thiele, Sauer, Atzmueller, and Kauffeld (2018a).

The main goal of this statistical technique is to model social dynamics from longitudinal network data. Observed changes between two or more networks can be described as functions of (1) individual characteristics of actors (e.g. variables such as age, gender or academic performance); (2) dyadic characteristics of actors (attributes linked to pairs of students, e.g. sharing the same academic interests or geographical origin); and (3) structural effects (linked to the network structure, e.g. reciprocity, network closure or cyclicity). In this research and according to our requirements, we make use of these three types of variables/effects to model the evolutionary patterns of students’ networks. The execution of the simulation model allows us to know which of these modelled variables/effects will support or inhibit the probability of one student sharing knowledge with another. The specific mathematical formulation of the simulation model and the specific set of effects that may be applied can be found in Snijders (2005).

Finally, it is important to note that in order to address the evolution of the influence of the proximity factors over the period studied, we specified the simulation model across the consecutive waves (i.e. from year 1 to year 2 and from year 2 to year 3).

### Operationalisation of the variables

Based on our theoretical framework, our SAOM analyses include the following variables and measures for each type of proximity dimension (Table 1).

**Table 1.**
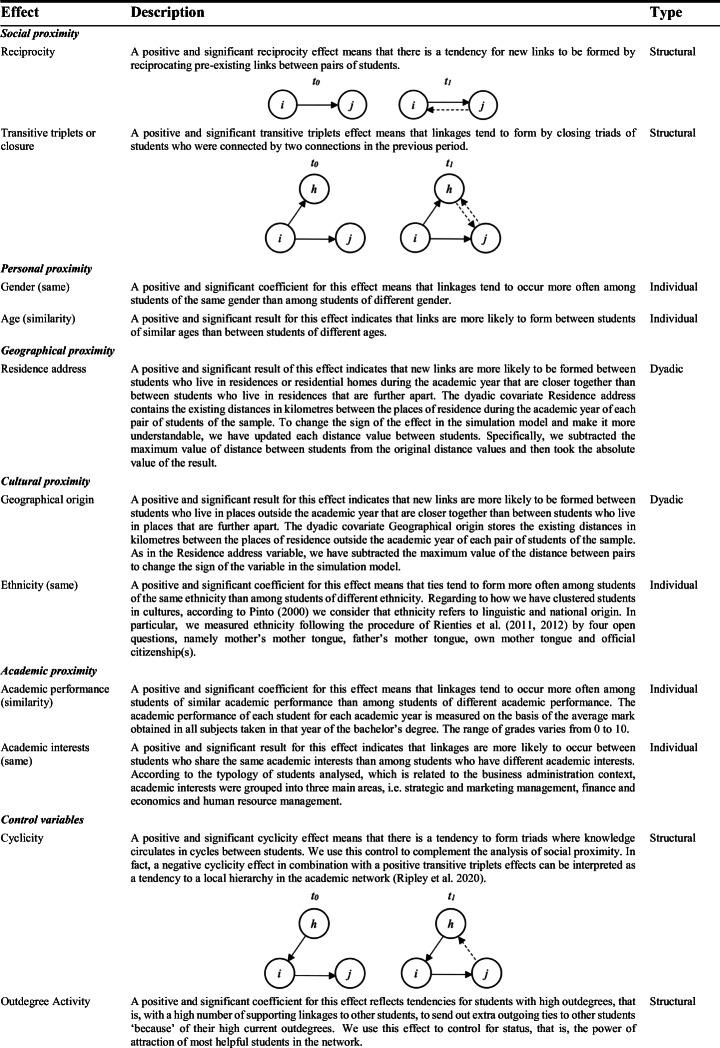

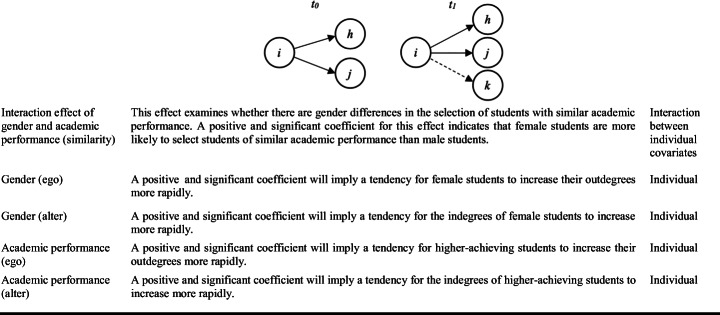
Summary of variables and measures for the SAOM analysis with RSiena

Note: For gender, female students were coded as 1 and male students as 0

As can be seen in Table 1, we have also included different control variables, which address aspects such as cyclicity, outdegree activity, the interaction effect of academic performance and gender on academic relationship establishment and the ego and alter effects for the gender and academic performance variables, in addition to the density and rate of change variables that are generally included to verify the validity of the SAOM.

## Results

### Descriptive analyses

Table 2 shows the descriptive statistics for the individual covariates of the sample of students addressed for each data collection point.

**Table 2.** Descriptive statistics for individual covariates

Variable	Wave 1		Wave 2		Wave 3	
	Mean/proportion	Std. dev.	Mean/proportion	Std. dev.	Mean/proportion	Std. dev.
Gender (prop. of female students)	49.30%	n.a.	51.32%	n.a.	51.32%	n.a.
Age	20.83	2.89	21.96	2.87	22.96	2.87
Ethnicity						
Spanish	74.65%	n.a.	76.32%	n.a.	76.32%	n.a.
Latin-American	15.49%	n.a.	14.47%	n.a.	14.47%	n.a.
Chinese	9.86%	n.a.	9.21%	n.a.	9.21%	n.a.
Academic performance	6.40	0.88	6.64	1.11	6.74	1.48
Academic interests						
Strategic and marketing management	35.21%	n.a.	38.16%	n.a.	43.42%	n.a.
Finance and economics	35.21%	n.a.	27.63%	n.a.	34.21%	n.a.
Human resource management	29.58%	n.a.	34.21%	n.a.	22.37%	n.a.

As follows from the data presented in the table, we can find a similar number of male and female students in the sample. On the other hand, the ethnic composition in the third year is 76.32% of Spanish students, 14.47% of Latin-American students and 9.21% of Chinese students. Non-Spanish students usually come from their country to complete their university studies at a European university. After completion they can decide to return to their country or continue their professional career in Europe. In relation to the mean academic achievement of the students in the sample, which ranges from 0 to 10 according to the Spanish education system, it has a value of 6.40 in the first year, with a slightly increasing trend over the period analysed. Finally, the academic area of interest in which the students analysed show most interest over time is strategic and marketing management.

In addition, Table 3 presents the data distribution of the dyadic covariates. As indicated in Table 1, the variable Residential address stores the existing distances in kilometres between the places of residence during the academic year of each pair of students while the variable Geographical origin contains the distances but between household locations (generally, family homes) outside the academic year. Since distance is a symmetric measure, the distribution of the distance between households is made between non-directed pairs of students. Based on the first wave, for n = 71 students there are n*(n−1) = 4,970 directed pairs that can be established. Therefore, if we consider non-directed student pairs, this figure must be halved, resulting in a total of 2,485 non-directed student pairs. For the second and third waves (n = 76 students), the number of non-directed student pairs is 2850. We have classified the non-directed students pairs in each wave for both dyadic covariates by distance ranges. The results show, as expected, that a large proportion of students’ households are only a few kilometres apart during the academic year. Specifically, approximately 51% of pairs are living at a distance smaller than 3 km. These values are similar for the 3 waves analysed. In contrast, outside the academic year, the percentage of households located at distances of less than 3 km is only 10%. On the other hand, about 70% of pairs are separated by 30 or more km. This is to be expected, as many students come from other counties, provinces and countries. As no student changed family residence during the three years of the study, the values of the dyadic covariate Geographical origin were the same for each wave, only excluding for the first wave, the data of the five students who entered the bachelor’s degree in the second year.

**Table 3.** Pairs of students classified by distance ranges for dyadic covariates

Distance range (km)	Residence address			Geographical origin		
	Wave 1	Wave 2	Wave 3	Wave 1	Wave 2	Wave 3
0.0–1.0	502	522	498	134	125	125
1.0–3.0	787	940	976	177	162	162
3.0–6.0	156	175	203	114	106	106
6.0–10.0	202	202	253	40	42	42
10.0–15.0	311	343	361	40	61	61
15.0–30.0	502	634	514	300	345	345
30.0–50.0	25	34	45	520	625	625
≥50.0	0	0	0	1160	1384	1384

Finally, Table 4 presents the descriptive statistics and the main changes in network composition for the three waves analysed. At first glance, it can be observed that students considerably develop academic relationships at university. This result is in line with previous evidence which also analysed academic linkages between university students (Chen et al., 2012; Tomás-Miquel et al., 2016). From a temporal point of view, the number of academic relationships increased significantly from the first to the second year, from 390 to 586 ties. In addition, the average degree, that is the average number of links developed per student, also increased from 5.493 to 7.711 in this period. On the other hand, from the second to the third year, these data stabilise without significant variations. These results make sense because during the first year at university students begin to get to know each other. However, it is not until the second and third year that they deepen their relationships. Regarding reciprocity and transitivity, the proportion of reciprocal relationships remains stable over time at around 60%, while the density of transitive triples in the network decreases from 55 to 42 %.

**Table 4.** Descriptive statistics and changes in composition of the academic network

Statistics	Wave 1	Wave 2	Wave 3
Nodes (students)	71	76	76
No. of ties	390	586	578
Average degree	5.493	7.711	7.605
Density	0.078	0.103	0.101
Isolated nodes	1	0	0
Reciprocity	60%	62%	60%
Transitivity	55%	49%	42%
Gini index	0.700	0.405	0.380
No. of ties created		306	168
No. of ties maintained from the previous wave		280	410
No. of ties dissolved from the previous wave		110	176

To gain a more accurate view of all these changes, we have additionally studied the existing concentration of support linkages between peers calculated using the Gini coefficient. This index allows us to explain whether some students develop more support relationships than others. It ranges from zero to one where zero means that there is a complete homogeneity in the number of links established per student and one the opposite. The results show the existence of a great heterogeneity in the formation of links in the first year of the students at university. However, this decreases significantly as the academic network evolves. Therefore, there is a tendency for all students to reduce over time the differences between them in the relational levels. These results are quite consistent because as time passes students can get to know each other better and develop more relationships, especially those less connected to the academic network in their early years at university. On the other hand, those students who developed many support links during the first and second years can become more selective in sharing knowledge, thus reducing the number of relationships developed.

Finally, it is also noteworthy to indicate that Jaccard Indices (0.38 from first to second wave and 0.54 from second to third wave), which are a measure for network stability, are all above 0.30 which are appropriate for this kind of dynamic analysis (Ripley et al., 2020). As suggested in other contributions such as Thiele, Sauer, Atzmueller, and Kauffeld (2018a), to deal with the differences in the number of actors in the sample between the different observations (71 students in the first year and 76 in the last two years), we used structural zeros.

### RSiena results

This section presents the empirical results obtained from the SAOM implemented in the RSiena package and attempts to answer the research questions proposed in the “Theoretical background and research questions” section.

Table 5 shows the results of the analyses in RSiena, including the influence of the analysed effects on the evolution of the academic network between the first and second year and also between the second and third year of the students at the university. For these SAOM analyses, parameters estimation was based on 2,406 iterations in both models. Basic rate parameters as well as convergence diagnostics, covariance and derivative matrices were based on 1,000 iterations for both analyses. Models convergence was good (*t*-ratios were lower than 0.09 for all coefficients in both models) and no important problems of multicollinearity were observed.

**Table 5.** Results of RSiena analyses

Variable	First-second grade			Second-third grade		
	Estimate	Std. error	*t*-value	Estimate	Std. error	*t*-value
Social proximity						
Reciprocity	1.599	0.201	7.971^***^	1.227	0.174	7.063^***^
Transitive triplets	0.349	0.042	8.399^***^	0.206	0.041	5.077^***^
Personal proximity						
Gender same	0.398	0.123	3.234^**^	0.510	0.140	3.651^***^
Age similarity	0.404	0.316	1.278	0.767	0.370	2.072^*^
Geographical proximity						
Residence address	0.034	0.010	3.304^**^	−0.010	0.009	−1.074
Cultural proximity						
Geographical origin	0.008	0.002	4.222^***^	0.001	0.002	0.389
Ethnicity same	0.366	0.178	2.057^*^	0.227	0.182	1.246
Academic proximity						
Academic performance (similarity)	0.665	0.299	2.227^*^	1.435	0.329	4.362^***^
Academic interests (same)	0.222	0.123	1.798	0.563	0.144	3.913^***^
Control variables						
Density	−2.076	0.223	−9.328^***^	−2.118	0.257	−8.257^***^
Cyclicity	−0.297	0.062	−4.770^***^	−0.181	0.064	−2.822^**^
Outdegree activity	−0.031	0.011	−2.748^**^	−0.026	0.013	−1.985^*^
Gender (*female*) ego	−0.121	0.164	−0.737	−0.038	0.170	−0.224
Gender (*female*) alter	−0.004	0.137	−0.031	0.264	0.145	1.818
Academic performance ego	−0.006	0.047	−0.129	0.056	0.052	1.077
Academic performance alter	−0.062	0.041	−1.521	0.017	0.049	0.351
Gender (female) x Academic performance (similarity)	−0.077	0.567	−0.135	0.890	0.657	1.353
Rate parameter (λ)	10.265	1.092		6.806	0.624	

Note: Significant at 0.05 level (^*^); 0.01 level (^**^); 0.001 level (^***^)

The rate of change and density parameters are normally included in this type of simulations. The former shows the probability of a student to establish new ties during the period as a measure of network change (Snijders, 2001). The latter represents the tendency of students not to establish academic links with just any other student on the network, that is, to exhibit a selective approach in reaching out to other peers (Ullrich et al., 2010). The rate of change parameter for the two periods analysed is positive and significant thus indicating a remarkable change in the formation of new ties. In addition, the density parameter in both models is negative and significant which is generally the case for social networks, except for contexts with extreme high densities (Balland, 2012).

To answer the first research question, the results of the influence of the effects associated to the different proximity factors in Table 5 are analysed. As can be seen, effects from the five proximity dimensions considered influence the formation of academic links between students at some point in the student’s career at the university. This confirms that the formation of academic relationships is a complex phenomenon that is induced by proximity factors from multiple areas.

On the other hand, and to answer the second research question, the influence of these factors over time is analysed. The results confirm that they do not influence with the same intensity for the different periods and dimensions. Therefore, both time and the proximity typology play an important role in the dynamics of academic relations.

We first find in Table 5 the effects of social proximity. As can be observed, it is the only dimension whose influence does not differ in the two time periods analysed. In this regard, the effects of reciprocity and transitive closure have a maximum and positive significance in the dynamics of establishing academic links both between first and second year and between second and third year.

For the rest of the proximity dimensions, they do not have the same influence in each of the periods analysed. In this way, we find important differences between periods for the effects of personal, geographical, cultural and academic proximity.

Regarding personal proximity, age similarity is not significant for the dynamics of creating links between the first and second year. However, between the second and third year this effect becomes significant. This result shows that although age similarity is not a relevant effect in the first two years at university, due to the fact that collaborative groups are often established at the beginning following more basic and observable criteria such as gender, residential proximity, geographical origin or ethnicity, with the passage of time, and after increasing mutual knowledge, it begins to play a significant role in the formation of academic links because of existing affinities between students of similar age, e.g. older students are more concerned about and involved with their families, while younger students are more concerned with campus activities (Graham & Donaldson, 1999). In addition, the effect of gender intensifies for the last two academic years. In this way, students show greater interest over time in developing relationships with others of the same gender.

On the other hand, the effects of geographical proximity are significant in the first period but lose their significance between the second and third year. Thus, although academic relations are based in part on the proximity of the students’ residences during their first stage at university because it facilitates a first contact between them, as students progress through university this effect weakens. This result is very relevant because it indicates that, although initially residential proximity can influence, as mutual knowledge among students increases this proximity is not “per se” sufficient, but rather it is necessary to share other traits and behaviours that facilitate knowledge exchange.

Another interesting result is related to cultural proximity. As can be seen in Table 5, the effect of geographical origin and ethnicity variables lose all significance in the last period analysed. This result is remarkable as it indicates how little relevance cultural proximity aspects have for the development of their academic networks once students progress through university.

Finally, we have found differences in the influence of the variables linked to academic proximity, although in opposite directions to previous dimension. In this sense, both the effects of similarity in academic performance and interests have intensified in the last two years. Therefore, students show greater interest over time in establishing links with students of similar academic performance and interests. This result is not surprising because these are aspects that require extensive experience and knowledge about peers to have an impact. In this way, it becomes difficult for freshman students to target other students with similar academic performance and interests because their personal network of relationships is very limited and there is not yet enough evidence of the performance or interests of other peers.

Regarding control variables, it should be noted that the effects of both cyclicity and outdegree activity weaken slightly over time, although still maintaining their significance. In general, the positive transitive triplets effect in combination with the negative cyclicity effect suggests the existence of local hierarchies with some students offering or receiving more support than others. On the other hand, and in the case of the outdegree activity effect, its negative effect indicates that the students who help the most during a given academic year show little willingness to establish new academic support links with other students during the following year. These results are in line with those obtained previously through the analysis of the evolution of the Gini coefficient. In this way, students become more selective in sharing knowledge due to the inherent costs of supporting processes that often arise in social contexts, including time and energy (Goodman & Darr, 1998). Regarding the ego and alter effects for gender and academic performance, none of them was significant. In the same vein, our results showed no gender differences in the selection of students with similar academic performance.

Further analysis and interpretation of all these results can provide important insights to help answer the third research question posed in the paper which aims to find a pattern that explains the uneven variation in the influence of the proximity dimensions throughout the period that students spend at university. In this respect, we can find a plausible explanation for this divergent evolution by grouping the proximity dimensions into three groups according to their level of influence over time.

A first group of factors would take into consideration those dimensions that facilitate the first contact between students and that help them to relate in the first years of university. In this period, the objective of many students would be to configure a first network of relationships as a social need, i.e. to feel integrated in a group. This set of factors would include geographical and cultural proximity which are dimensions linked to spatial proximity and/or characteristics that are easily perceived among students, such as ethnicity, and which facilitate first contact and mutual relationship. We refer to them as shallow or early proximity factors. As students progress through university, the capacity of these factors to explain the formation of academic links falls dramatically. These reflections are in line with the suggestions of Mayer and Puller (2008) who indicate that although observable characteristics such as race clearly play a role in relationship formation, they have limited explanatory power for the formation of bonds among students at university. Similarly, Preciado et al. (2012) in environments of adolescent friendships also demonstrate the reduction of the influence over time of aspects such as ethnicity in the formation of bonds. On the contrary, our results are not consistent with those offered by other contributions that highlight the extreme influence that residential proximity has on the formation of friendship networks in contexts of secondary education or relationships between adults such as Tsai (2006) or Preciado et al. (2012). This divergence may help appreciate the particularities of academic networks in universities and the need to carry out specific studies such as the present research.

A second set of factors would group together those dimensions linked to strategic aspects through which students optimise their relationships from their second year at university. This group would include the dimensions of academic and personal proximity. We can refer to them as deep or late proximity factors. As mentioned above, support processes represent a waste of energy and time, among other aspects, for helpful students. Therefore, as our results have shown, they become more selective over time in the formation of their academic links. In this regard, from the establishment of a first network of relationships and increased knowledge of peers, our results show that from the second year, students will strategically seek to prioritise the establishment of relationships with those peers of similar academic performance (a strategy especially developed by higher-performing students), or similar academic areas of interest in order to pave the way for the improvement of their performance and the achievement of their academic goals. In the same way, they will also prioritise the formation of links with students of similar gender and age. These personal similarities will facilitate communication between these students and improve mutual understanding of their academic needs and expectations. All these results are fairly consistent with previous contributions such as Ullrich et al. (2010), Thiele, Sauer, Atzmueller, and Kauffeld (2018a) or Brouwer et al. (2018) which highlight the relevance that the similarity in gender and age exerts in the formation of academic linkages at university throughout the different academic years. In addition, they are also in line with the theoretical assumptions proposed in Thiele, Sauer, Atzmueller, and Kauffeld (2018a) who argue that students’ decisions for linkage formation in the classroom follow more strategic decision patterns over time.

Finally, the last group would include social proximity, which is a mechanism that influences with the same intensity the dynamics of academic links in all academic years. Therefore, we can consider it as a stable or permanent proximity factor. Mayer and Puller (2008) show that common friends are a good predictor of the existence of a relationship between two students. In this sense, the trust that comes from having a mutual friend facilitates knowledge transmission and academic support. Moreover, the prior receipt of academic support from a student also facilitates the subsequent presence of a reciprocal response, thus consolidating the relationship. Students use social proximity to extend and consolidate their network of academic contacts. In this regard, in the initial years at university and from a social need for integration into the group, they can use social proximity to expand their network of academic relations. This becomes evident from the increase in the average degree per student that takes place between the first and second year, as shown in Table 4. In the final years, social proximity would also be a key factor for students as it would allow them to get closer through their existing network of academic relationships to new students who are more similar in personal and academic aspects.

## Conclusions

This research has focused from a multidimensional perspective on the complex factors that influence the configuration of academic networks among undergraduate students at university. In particular, we have tried to understand which proximity dimensions influence the formation of these relationships. Furthermore, we have also explored the influence that these proximity dimensions have throughout the entire time a student spends at university.

On a general level, the results indicate that students’ academic networks at university differ significantly from the networks that would arise from random peer selection. Therefore, they are neither homogeneous nor stable, but rather cliquish and dynamic with a continuous evolution of their structure throughout the years that students remain on campus. In addition, the results have also shown that the formation of academic relationships is derived, in part, from a wide range of proximity factors of a social, personal, geographical, cultural and academic nature, although with an uneven influence throughout the student’s progression through university.

A deeper analysis of the results has allowed us to arrange the proximity factors in three groups, according to their level of influence over time. This has made it possible to especially highlight the relevant role that personal, academic and social proximities play in the formation of solid relationships during the final years at the university. In particular, we propose that the similarity in personal factors such as ethnicity, geographical origin or residential proximity is crucial for the formation of academic exchange relationships in the student’s early years at university. However, as time passes, the influence of these proximities disappears and the search for supportive peers becomes mostly determined by more strategic aspects for the student such as the establishment of relationships with other students of the same gender or similar academic performance, interests or age.

The results obtained are fairly consistent with past contributions which had confirmed the existence of a specific set of factors that influence the formation of academic linkages in university contexts (Brouwer et al., 2018; Thiele, Sauer, Atzmueller, & Kauffeld, 2018a). However, despite these past efforts, our research has gone one step further as these previous works have only focused on limited time periods and factors to analyse the formation of bonds between undergraduate students. On this basis, we consider that there are two main implications of our work for educational research. In this regard, this research allows to improve the comprehension in a single context of study of the dynamics of knowledge exchange and academic support linkages between undergraduate students from an alternative, comprehensive, integrated and enriching framework of proximity factors. In this sense, our results may pave the way and encourage researchers to the development of new contributions which deepen and extend the framework proposed to better understand the processes of formation of student relationships in higher education. On the other hand, while we have no doubt about the influence of different factors on the formation of academic links between students, the influence of time should not be overlooked. As our results reveal, the specific stage in which the student is at the university is fundamental to understanding the dynamics of academic ties.

Complementing the theoretical contribution, this research also provides diverse implications for educators and educational policy and strategy. In this regard, our results improve the understanding of social dynamics between undergraduate students which might help guide universities on the development of strategies aimed at improving the management of work groups both at a general level and at the level of the classroom itself. In our opinion and based on our results, educators should reduce the temptation to group students according to aspects of geographical, ethnic or cultural proximity as these are transitory and non-strategic proximity aspects for students. On the other hand, the preference of students for others with similar personal and academic characteristics to establish academic relationships may lead in the final years at the university to the emergence of working groups excessively polarised in terms of age, gender and academic interests and performance which may not resemble the working environments they will encounter once they enter the labour market. This can lead to even greater disparities in performance between more and less advanced students, possibly causing the latter to become demotivated and drop out, especially those with less emotional skills. In this regard and in line with the proposals of other researchers such as Furmedge et al. (2014) or Brouwer et al. (2018), educational policymakers should promote the inclusion of peer-assisted learning through small learning groups to motivate less and more advanced students to cooperate with each other and thus reducing the segregation and the presence of polarised learning groups. Furthermore, the tendency towards the formation of student peer groups who are relatively similar in terms of academic interests, while facilitating their communication and mutual understanding, may hinder creativity and innovation. In this sense, as suggested by authors such as Tomás-Miquel et al. (2016), Zhang and Huai (2016) or Thiele, Sauer, Atzmueller, and Kauffeld (2018a), the formation of heterogeneous working groups in terms of knowledge and skills is crucial to promote their innovation and performance. We therefore suggest that universities should be aware of these trends and move towards an organisation of working groups that allows for the presence of students with different academic skills and interests to maximise their effectiveness in teamwork.

Finally, this research also suffers from certain limitations. Firstly, we have not included in the study different predictors of academic relationship establishment, such as college experience, socioeconomic status or career aspirations which would have enriched our model. We have also not considered in our framework the inclusion of proximities between students in personality and emotional traits such as extraversion, responsibility, agreeableness or self-confidence, which has already been addressed in recent contributions such as Thiele, Sauer, and Kauffeld (2018b). Although personality aspects are somewhat complex to measure, they can certainly bring interesting new perspectives to the measures of personal proximity. Secondly, we have developed our study in a single study program and cohort. Therefore, generalisations in other contexts should be made with caution. On the other hand, although the sample is gender-balanced, in other aspects such as ethnicity, its diversity is quite limited, so our results may vary in other regions, countries or cultures. Therefore, future research should further investigate and replicate our results using a more diverse sample concerning culture, cohorts and characteristics of the study program. Thirdly, it is important to consider how lecturers allocate students into the respective cohorts/subgroups/teams/tasks and the influence this may have on the establishment of future academic links, as suggested by contributions such as Rienties and Nolan (2014) or Rienties and Héliot (2018). Although in our study program lecturers usually give students the freedom to organise themselves into groups, its influence should not be underestimated. Fourthly, as several covariates used are time-dependent, we could have opted to employ the extended SAOM for the co-evolution of networks and behaviours (Snijders et al., 2010). Finally, our results have been obtained in a context of mainly face-to-face education, where students have been able to be in close contact for years. The new challenges that the COVID-19 pandemic has demanded from higher education, such as the change of most of the courses to online mode, will undoubtedly condition the formation of academic links between students in the face of a clear lack of face-to-face contact. Therefore, the proposed framework may not be appropriate in this context, where a priori new proximities may appear, such as similarities in technological skills, and others further reduce their influence, such as geographical closeness.

In spite of these limitations, which can be addressed in future research, this paper has shed light on the complex dynamics of academic relations between university students by offering a novel framework for time-dependent analysis of the influence of proximity factors. In this respect, we are particularly convinced that it can serve as a basis for the dynamic study of the influence of new proximity dimensions, which could be classified according to their early, late or permanent impact on the formation of academic linkages among students throughout their university years, thus opening a promising research area.
